# Supplementary material for: A deep learning system for heart failure mortality prediction
Source: PLoS One. 2023 Feb 24;18(2):e0276835. doi: 10.1371/journal.pone.0276835 (PMC9956019; doi:10.1371/journal.pone.0276835)
Supplement: S1 File — (DOCX) [file pone.0276835.s002.docx]

**Appendix**

**Appendix A. There are 26 major categories of diseases and the sub-categories diseases included**

| **Major diseases** | **Sub-categories diseases** | **Major diseases** | **Sub-categories diseases** |
| --- | --- | --- | --- |
| Cardiac arrhythmias | Atrioventricular block etc. | Cardiomyopathy | Alcoholic cardiomyopathy etc. |
| Valve disease | Mitral stenosis etc. | Myocarditis | Idiopathic myocarditis etc. |
| Endocarditis | Gonococcal endocarditis etc. | Pericardial disease | Cardiac tamponade etc. |
| Pulmonary circulation disorder | Chronic pulmonary embolism etc. | Coronary atherosclerosis I | Postmyocardial infarction syndrome etc. |
| Respiratory failure | Acute respiratory failure etc. | Diabetes | Secondary diabetes mellitus etc. |
| Peripheral vascular disorder | Atherosclerosis of renal artery etc. | Coronary atherosclerosis II | Intermediate coronary syndrome etc. |
| Hypertension | Benign essential hypertension etc. | Renal failure | Acute kidney failure, unspecified etc. |
| Renal disease | Diabetes insipidus etc. | Hyperlipidemia | Lipoprotein deficiencies etc. |
| Abnormal heart structure | Ventricular septal defect etc. | Connective tissue disease | Lupus erythematosus etc. |
| Obesity | Morbid obesity etc. | Hyperthyroidism | Thyrotoxicosis from ectopic thyroid nodule etc. |
| Alcohol abuse | Alcohol withdrawal etc. | Drug abuse | Opioid type dependence etc. |
| Cardiac Arrest and Sudden Cardiac Death Disease | Cardiac arrest etc. | Congenital cardiovascular disorders | Tetralogy of fallot etc. |
| Tobacco | Tobacco use disorder etc. | Other heart Disease | Kyphoscoliotic heart disease etc. |

**Appendix B. There are 22 laboratory tests**

| **Name** | **Unit** | **Name** | **Unit** |
| --- | --- | --- | --- |
| White cells count (WBC) | K/uL | [Urea](javascript:;) [nitrogen](javascript:;) (BUN) | mg/dL |
| Hemoglobin | g/dL | Kalium (K) | mEq/L |
| Hematocrit | % | Sodium (Na) | mEq/L |
| [Blood](javascript:;) [platelet](javascript:;) [count](javascript:;) (PLT) | K/uL | Chloridion (Cl) | mEq/L |
| Blood glucose (GLU) | mg/dL | Hormothyrin (TSH) | uIU/mL |
| [Albumin](javascript:;) (ALB) | IU/L | Troponin T (cTnT) | ng/mL |
| Total bilirubin (TBIL) | mg/dL | Creatine kinase (CK) | IU/L |
| Alanine transaminase (ALT) | IU/L | Creatine kinase isoenzyme (CK-MB) | IU/L |
| [Aspartate](javascript:;) [aminotransferase](javascript:;) (AST) | IU/L | Antithrombin | Seconds |
| [Alkaline](javascript:;) [phosphatase](javascript:;) (ALP) | IU/L | [Arterial](javascript:;) [blood](javascript:;) [gas](javascript:;) [analysis](javascript:;) (pO2) | mm Hg |
| [Serum](javascript:;) [creatinine](javascript:;) (CRE) | mg/dL | [Arterial](javascript:;) [blood](javascript:;) [gas](javascript:;) [analysis](javascript:;) (pCO2) | mm Hg |
