# Supplementary material for: A deep learning system for heart failure mortality prediction
Source: PLoS One. 2023 Feb 24;18(2):e0276835. doi: 10.1371/journal.pone.0276835 (PMC9956019; doi:10.1371/journal.pone.0276835)
Supplement: Attachment — Submitted filename: Response to Reviewers.docx [file pone.0276835.s003.docx]

Dear editor,

Thank you very much for your valuable suggestions on our paper. We have complied with journal requirements and revised these opinions one by one at the first time. In the revised manuscript with track changes file, the revision has been marked in yellow. Please review the following the modification notes for each journal requirement and suggestion. The comment is blue and response is black.

Journal Requirement: 1

Please ensure that your manuscript meets PLOS ONE's style requirements, including those for file naming.

Thanks very much for reminding the style requirements. We have carefully modified the article format according to the given files.

Journal Requirement: 2

We suggest you thoroughly copyedit your manuscript for language usage, spelling, and grammar.

Thanks very much for your suggestion about copyediting our manuscript for language usage, spelling, and grammar. We have carefully checked the grammar, spelling and other problems of the paper.

Journal Requirement: 3

Thank you for stating the following financial disclosure:

The article is supported by the following projects. (1) National Major Scientific The General Object of National; Natural Science Foundation (62076177、61772358). (2) National Major Scientific Research Instrument Development Project (6202780085). (3) Shanxi Province key technology and generic technology R&D project (2020XXX007).

Please state what role the funders took in the study. If the funders had no role, please state: "The funders had no role in study design, data collection and analysis, decision to publish, or preparation of the manuscript." If this statement is not correct you must amend it as needed.

Please include this amended Role of Funder statement in your cover letter; we will change the online submission form on your behalf.

Thanks very much for your reminding us to state what role the funders took in the study. Financial help is provided by this funder. And this research topic comes from the National Natural Science Foundation of China 62076177. We have included this funder statement in our cover letter. Please help me to change the online submission.

Journal Requirement: 4

In your Data Availability statement, you have not specified where the minimal data set underlying the results described in your manuscript can be found. PLOS defines a study's minimal data set as the underlying data used to reach the conclusions drawn in the manuscript and any additional data required to replicate the reported study findings in their entirety. All PLOS journals require that the minimal data set be made fully available. For more information about our data policy, please see <http://journals.plos.org/plosone/s/data-availability>.

Upon re-submitting your revised manuscript, please upload your study’s minimal underlying data set as either Supporting Information files or to a stable, public repository and include the relevant URLs, DOIs, or accession numbers within your revised cover letter. For a list of acceptable repositories, please see http://journals.plos.org/plosone/s/data-availability#loc-recommended-repositories. Any potentially identifying patient information must be fully anonymized.

Important: If there are ethical or legal restrictions to sharing your data publicly, please explain these restrictions in detail. Please see our guidelines for more information on what we consider unacceptable restrictions to publicly sharing data: http://journals.plos.org/plosone/s/data-availability#loc-unacceptable-data-access-restrictions. Note that it is not acceptable for the authors to be the sole named individuals responsible for ensuring data access.

We will update your Data Availability statement to reflect the information you provide in your cover letter.

First of all, our data is not available upon request from the MIT Laboratory for Computational Physiology Institutional Data Access / Ethics Committee.

Our data is from the MIMIC-III (Medical Information Mart for Intensive Care III) belongs to the MIT Laboratory for Computational Physiology Institutional Data Access / Ethics Committee. We obtain permission to use MIMIC-III according to the requirements and downloaded the corresponding data. But we don't have the authority to make that data public. In the <https://mimic.mit.edu/docs/gettingstarted/> website, the data is a restricted-access resource. All who want to use the data must be a [credentialed user](https://physionet.org/settings/credentialing/) and [sign the data use agreement](https://physionet.org/sign-dua/mimiciii/1.4/) for the project from <https://physionet.org/> website. In order to show that, we intercepted part of the website information, as shown in the Fig 1.

**Fig 1. The screenshot from** [**https://mimic.mit.edu/docs/gettingstarted/**](https://mimic.mit.edu/docs/gettingstarted/) **website**

In addition we find the email between the data authorization and us. Fig 2 is a partial screenshot from one of the emails. It shows the data use agreement and the statement 3 framed in red means that we don't have the authority to make that data public.

**Fig 2. The email information between the data authorization and us.**

In order to meet the data requirements, we give all necessary steps for getting access MIMIC-III permissions as follows.

In the first step, login to the **CITI program** website <https://about.citiprogram.org/>, fill in the information as required and register the user. The CITI program home page is shown in Fig 3. When you complete the registration, you will receive an email containing your username and initial password.

**Fig 3. The home page of CITI program.**

In the second step, login the CITI program. Then take the exam under *Data or Specimens Only Research* and ensure an average score of 90 or more on the exam. The Fig 4 and Fig 5 show how to find the courses. Fig 6 shows there are nine exams you need to accomplish.

**Fig 4. The courses we need to accomplish.**

**Fig 5. Entering the courses.**

**Fig 6. There are nine exams need to be accomplished.**

In the third step, obtain the CITI gradebook and completion record as shown in Fig 7.

**Fig 7. Download the CITI gradebook and completion record.**

In the fourth step, login to the PhysioNet website <https://physionet.org>, fill in the information as required and register the user.

**Fig 8. Using an email to login to PhysioNet.**

In the fifth step, submit gradebook obtained in the third step and apply for permission to use MIMIC-IIII. After submitting your application, the data authorization will ask you to fill your colleagues or co-authors. And the data authorization will ask if they are familiar with your project or if the database should be open to you. Then your colleagues or co-authors according to the requirement [respond](javascript:;) [to](javascript:;) the [email](javascript:;). Through a few week-days, if you get approved you will get an email shown in **Fig 2.**

The above is all necessary instructions required for users to become credentialed and sign the data use agreement. After that, a user can login to the MIMIC website and download the MIMIC-III data.

MIMIC-III includes 26 tables and the data can be shown by PostgreSQL database systems. And we utilize the SQL code to view and extract data about heart failure patients. According to the instruction from <https://journals.plos.org/plosone/s/data-availability> website, we could provide data extraction code, which is packed and upload as a supportable file. In addition, we uploaded our code through GitHub <https://github.com/foneone/SQL-code-for-extracting-heart-failure-from-MIMIC>. All the authorized users of MIMIC-III can reference the website to extract related data.

According to new data requirement letter, we revise the original cover letter and add new data availability statement in the cover letter.

Reviewer #1

In the present study, the authors presented a heart failure mortality prediction model using machine learning, which inspired many researchers. There are many models that predict the prognosis of heart failure by conventional statistical methods and currently, new predicting models using deep-learning algorithms have been introduced showing outstanding performance. In this study, the authors proposed an indicator vector to indicate whether the value is true or be padded, which fast solves the missing values and helps expand date dimensions. It appears that the study has been carefully done and the manuscript is well written and clearly presented. However, the following issues require further consideration and clarification.

Thanks very much for your valuable comments and reminding. Sincerely appreciate your recognition of our work. We replied your comments one by one and revised the whole paper carefully.

Reviewer #1: 1)

First, authors should describe the development of machine learning models in detail enough for readers to reproduce the experiment.

This comment mainly involves the section of *CNN with different kernel sizes.* In order to explain the CNN model framework used by us, we revise Figure 3 in the original paper which is shown as Fig 9 in this paper.

As showing in Fig 9, the CNN structure includes seven parts. We explain each part in detail as follows. Firstly, the input structure is 256*(1*90). The 256 is the batch size, which means CNN framework can handle 256 pieces of data at one time. We obtain 90 features of heart failure patient from MIMIC-III database. Although the CNN model in this study uses 1D convolution, the CNN model based on pytroch framework needs the two-dimensional input format. Thus, we expand the original dimension and the input becomes 1*90, which is a two-dimensional format.

Secondly, the input will go through the convolution layer, which is purple structure shown in the Fig 9. The convolutional layer contains three parts. First of all we used eight convolution kernels of the same size (1*3). And then we use the batch normalization (BN) structure to avoid the gradient disappearance and explosion phenomenon problems. The BN accelerates simultaneously the convergence rate. At last the RuLU function is chosen as an activation function. And the stride is set to 1 and padding is set to 1 in the convolutional kernel structure.

**Fig 9. The modificated CNN framework based on multi-head self-attention.**

Thirdly, after the convolution layer, the data goes through the different kernels layer. The different kernels layer contains three different type groups by the kernel size. The red kernel groups contain three convolution kernels of the same size (1*3). And then the red kernel groups also include BN and ReLU activation function. At last we put the Maxpooling structure behind the ReLU function. The Maxpooling’s sampling window is set to 2 and other parameters in Maxplooing are default. Similar to the red kernel groups, the only difference in blue kernel groups is that the kernel size is (1*4) and the padding is set to 1. And the only difference in orange kernel groups is the kernel size is (1*5) and padding is set to 1compared to the red kernel groups. The other parameters in blue and orange kernel groups are the same as the red kernel groups.

Fourthly, after the data goes through the different kernels layer, the output becomes 256*(3*44), 256*(3*44) and 256*(3*44). The stitching layer needs to splice three outputs and after that the data becomes 256*(3*132). Fifthly, the data goes through the multi-head self-attention mechanism which is introduced in detail in the section of *Multi-head self-attention mechanism.* The mechanism has only one parameter is the number of head. And in this study, we set the head of mechanism is 2.

Sixthly, the data goes through the fully connected layer, which is grey in Fig 9. The fully connected layer has two fully connected layers: (396*128) and (128*2). Finally, the output is 256*2 after the fully connected layer. Therefore we gain 256 predictions about the heart failure patients are dead or alive.

The above is a detailed introduction of the CNN model. In order to display model parameters more clearly, we draw the Table 1 as follows. Following the Table 1, the readers can easily construct our CNN model. And we also revise the relevant content in the original paper.

**Table 1. The parameters of CNN model in this study**

| **Number** | **Name** | **Sizes** | **Output** |
| --- | --- | --- | --- |
| 1 | Input | 256*(1*90) | - |
| 2 | Convolution layer | Conv1d(1,8,3,1,1); BatchNorm1d(8); ReLU | 256*(8*90) |
| 3 | Different kernels layer | Conv1d(8,3,3,1,0); BatchNorm1d(3); ReLU; MaxPool1d(2) | 256*(3*44) |
|  |  | Conv1d(8,3,4,1,1); BatchNorm1d(3); ReLU; MaxPool1d(2) | 256*(3*44) |
|  |  | Conv1d(8,3,5,1,1); BatchNorm1d(3); ReLU; MaxPool1d(2) | 256*(3*44) |
| 4 | Stitching layer | - | 256*(3*132) |
| 5 | Multi-head self-attention | 2 (the head) | 256*(3*132) |
| 6 | Fully connected layer | (396, 128) and (128,2) | 256*2 |
| 7 | Output (Results) | - | 256*2 |

Reviewer #1: 2)

As a major pitfall of machine learning (ML) algorithms is overfitting, external validation is needed. Without external validation, the study result is not guaranteed in other hospitals.

We quite agree with this comment that machine learning (ML) algorithms often have the problem of overfitting. We will analyze and solve the over-fitting problem as follows.

To begin with in order to validate our model, we apply the proposed model to other data except MIMIC-III. Because of the data privacy, it is hard to find the data about dead or alive heart failure patients. Even if there is public available data on the heart failure patients, the public data rarely has as many patient characteristics as MIMIC-III database. Finally, we find a dataset published on December 1, 2020 with a large number of features.

The dataset comes from a Zigong Fourth People’s Hospital in Sichuan, China between 2016 and 2019, including 168 variables for patients with heart failure^[1, 2]^. In order to distinguish this dataset from MIMIC-III database, we name this dataset HFP-ZGFPH(**H**eart **F**ailure **P**atients in **Z**i**g**ong **F**ourth **P**eople’s **H**ospital). The HFP-ZGFPH database is a restricted-access resource and we can request access via <https://physionet.org/content/heart-failure-zigong/1.2/>.

In HFP-ZGFPH database, 2008 patients have token medicine. Medical records are shown in dat_md.csv. There are 18 medicines in HFP-ZGFPH database. In the original paper, we divide medicines into seven categories: ACEI, ARB, beta-receptor blockers, CCB, digitalis, diuretic and nitrates. According to such classification rules, we also classify 18 medicines into categories. The detail is shown in Table 2.

**Table 2. The category of 18 medicines in HF HFP-ZGFPH database**

| **Name** | **Category** | **Name** | **Category** |
| --- | --- | --- | --- |
| Sulfotanshinone sodium injection | None of category | Ddigoxin tablet | Digitalis |
| Furosemide tablet | Diuretic | Hydrochlorothiazide tablet | Diuretic |
| Torvastatin calcium table | None of category | Spironolactone tablet | Diuretic |
| Furosemide injection | Diuretic | Valsartan dispersible tablet | ARB |
| Milrinone injection | Noe of category | Deslanoside injection | Digitalis |
| Torasemide tablet | Diuretic | Nitroglycerin injection | Nitrates |
| Benazepril hydrochloride tablet | ACEI | Shenfu injection | None of category |
| Torvastatin calcium table | None of category | Spironolactone tablet | Diuretic |
| Dobutamine hydrochloride injection | None of category | Isoprenaline hydrochloride injection | Beta adrenoceptor agonist |

Because we only consider seven categories of drugs in the original paper. Therefore, regardless of how many different diuretics a patient is taking, we think he is taking diuretics. That is to say, if a patient has both took furosemide tablet and spironolactone tablet, we mark the patient as taking diuretics. Furthermore, there are 7 medicines not belonging to the seven categories. We do not think about these medicines. It is particularly emphasized that the receptor drugs in the article are beta receptor blockers, while isoproterenol hydrochloride injection in the database is a beta adrenoceptor agonist drug, so it is not included in the 7 categories. Therefore, HFP-ZGFPH database’s 18 medicines are converted to seven drugs as medicine characteristics of patient shown in Table 3.

According to the feature classification method and representation method in Table 3 which is in the original paper, the features in HFP-ZGFPH are described in Table 3. In Table 3, the index-M is the feature index in the original paper. And the index-H represents the characteristic index in the data dictionary table of the HFP-ZGFPH database. As well as, the Feature name-M is the feature meaning in HFP-ZGFPH database. The Feature name-M is the corresponding feature name in the original paper. We also adopt one-hot coding rule of features. For example, digit 0 denotes male and digit 1 denotes female according to the one-hot coding rule in the original paper. The one-hot coding rule of medication and related diseases is same as the original paper.

**Table 3. The features in FIC database**

| **Index-M** | **Index-H** | **Classification** | **Feature name-H** | **Feature name-M** | **Representation** | **Meaning and scope (unit)** |
| --- | --- | --- | --- | --- | --- | --- |
| 1 | 8 | Gender | Gender | Gender | One-hot coding | 0 means male, 1 means female |
| 2 | 166 | Age | Agecat | Age | Value | The median of patient's age range |
| 3-9 | - | Medication | The corresponding relationship is shown in Table 2. | ACEI | One-hot coding | 1 means used, 0 means not. |
|  |  |  |  | ARB |  |  |
|  |  |  |  | Beta-receptor blockers |  |  |
|  |  |  |  | CCB |  |  |
|  |  |  |  | Digitalis |  |  |
|  |  |  |  | Diuretic |  |  |
|  |  |  |  | Nitrates |  |  |
| 10-13 | - | Surgery | This information does not exist. | LVAD | One-hot coding | Because the feature is not existed. So we use {0,0,0,0} to fill the feature. |
|  |  |  |  | CRT |  |  |
|  |  |  |  | ICD |  |  |
|  |  |  |  | Heart transplantation |  |  |
| 14-39 | 26 | Related diseases | Chronic obstructive pulmonary disease | Pulmonary circulation disorder | One-hot coding | 1 means with this related diseases, 0 means not. Other disease features (totally 10) that do not exist are filled with digit 0. |
|  | 38 |  | type II respiratory failure | Respiratory failure |  |  |
|  | 23 |  | Peripheral vascular disease | Peripheral vascular disorder |  |  |
|  | 12-13 |  | Systolic blood pressure  diastolic blood pressure | Hypertension |  |  |
|  | 30 |  | Moderate to severe chronic kidney disease | Renal disease |  |  |
|  | 17 |  | BMI$\geq$28 | Obesity |  |  |
|  | 21 |  | Myocardial infarction | Coronary atherosclerosis I |  |  |
|  | 29 |  | Diabetes | Diabetes |  |  |
|  | 46 |  | Acute renal failure | Renal failure |  |  |
|  | 27 |  | Connective tissue disease | Connective tissue disease |  |  |
| 40 | - | ICU turnover times | This information does not exist. | ICU turnover times | Value | Because the feature is not existed. So we use {0,0,0} to fill the feature. |
| 41 |  | ICU stay time |  | ICU stay time | Value |  |
| 42 |  | Stayed in CCU |  | Stayed in CCU | One-hot coding |  |
| 43-86 | 70 | Laboratory test | White blood cell | White cells count (WBC) | Value | *10^9/L |
|  | 86 |  | Hemoglobin | Hemoglobin |  | g/L |
|  | 77 |  | Hematocrit | Hematocrit |  | % |
|  | 87 |  | Platelet | [Blood](javascript:;) [platelet](javascript:;) [count](javascript:;) (PLT) |  | *10^9/L |
|  | 152 |  | Glucose blood gas | Blood glucose (GLU) |  | mmol/l |
|  | 119 |  | Albumin | [Albumin](javascript:;) (ALB) |  | g/L |
|  | 129 |  | Total bilirubin | Total bilirubin (TBIL) |  | umol/l |
|  | 124 |  | Glutamic pyruvic transaminase | Alanine transaminase (ALT) |  | U/L |

**Table 3. The features in FIC database (continued)**

|  | - |  | - | [Aspartate](javascript:;) [aminotransferase](javascript:;) (AST) |  | - |
| --- | --- | --- | --- | --- | --- | --- |
|  | 126 |  | Alkaline phosphatase | [Alkaline](javascript:;) [phosphatase](javascript:;) (ALP) |  | (U/L) |
|  | 65 |  | Creatinine enzymatic method | [Serum](javascript:;) [creatinine](javascript:;) (CRE) |  | umol/L |
|  | 66 |  | Urea | [Urea](javascript:;) [nitrogen](javascript:;) (BUN) |  | mmol/l |
|  | 149 |  | Potassium ion | Kalium (K) |  | mmol/l |
|  | 150 |  | Chloride ion | Sodium (Na) |  | mmol/l |
|  | 151 |  | Sodium ion | Chloridion (Cl) |  | mmol/l |
|  | - |  | - | Hormothyrin (TSH) |  | - |
|  | 100 |  | High sensitivity troponin | Troponin T (cTnT) |  | pg/mL |
|  | 112 |  | Creatine kinase | Creatine kinase (CK) |  | IU/L |
|  | 113 |  | Creatine kinase isoenzyme | Creatine kinase isoenzyme (CK-MB) |  | IU/L |
|  | - |  | - | Antithrombin |  | Because the feature is not existed. |
|  |  |  |  | [Arterial](javascript:;) [blood](javascript:;) [gas](javascript:;) [analysis](javascript:;) (pO2) |  |  |
|  |  |  |  | [Arterial](javascript:;) [blood](javascript:;) [gas](javascript:;) [analysis](javascript:;) (pCO2) |  |  |
| 87-88 | - | Heart rate | Pulse rate | Heart rate | Value | beats/s |
| 89-90 | 17 | BMI | BMI | BMI | Value | (kg/m^2) |

**Table 4．Unit conversion table**

| **Value (unit) in HFP-ZGFPH** | **Value (unit) in MIMIC-III** | [**Remark**](javascript:;) |
| --- | --- | --- |
| 1 (*${10}^{9}$/L) | 1 (K/uL) | WBC, PLT |
| 1 (g/L) | 1*${10}^{-1}$ (g/dL) | Hemoglobin |
| g/L | IU/L | No conversion relationship (ALB) |
| 1 (umol/L) | 0.011312 (mg/dL) | TBIL |
| 1 (mmol/L) | 18 (mg/dL) | GLU |
| U/L | IU/L | No conversion relationship |
| 1 ( mmol/L) | 1 ( mEq/L) | K, Na, Cl |

However, some features in HFP-ZGFPH database do not exactly match features in MIMIC-III. Therefore, in order to make use of features in HFP-ZGFPH database, we change some features. The age in HFP-ZGFPH database is not a specific age and just is an age range. But the age of the original paper is a specific age. So we take the median of each patient's age range to represent the patient's age. In addition, HFP-ZGFPH database does not record hypertension as a related disease. But every patient has blood pressure testing. Thus, we use systolic blood pressure and diastolic blood pressure to predict whether a patient has high hypertension. Obesity as a related disease is the same as hypertension. We use BMI to predict whether a patient has high obesity. The classification of laboratory test has the problem of disunity of units of measurement. So we unify the units according to the units of measurement in the original text. And the unit conversion table is shown in Table 4. Moreover, HFP-ZGFPH database does not include the feature of heart rate. We use the pulse rate instead. Even though we know it's not accurate to substitute pulse rate for heart rate, in order to make the most of the feature and not waste the feature, we have to substitute the pulse rate for heart rate. The laboratory test of [Urea](javascript:;) [nitrogen](javascript:;) (BUN) and Troponin T (cTnT) is also not in the HFP-ZGFPH database. For avoiding high feature loss rate, we use Urea and High sensitivity troponin instead of BUN and cTnT.

Inevitably, feature loss and single sample loss rate are high in the HFP-ZGFPH database. Table 3 contains 24 variables not existing in HFP-ZGFPH database. If we treat all the 24 features as missing features, the number of samples available for the experiment is 0. Thus, we consider the 10 missing features in related diseases are normal. That is to say, all patients have not 10 related diseases. On this basis, we remove samples with feature loss rate of a single sample higher than 30%. The feature missing problem is dealt with by using the method of feature missing value processing in the original paper.

The HFP-ZGFPH database record four types of mortality: survivable patients, dead within 28 days patients, dead within 90 days patients, dead within 180 days patients. In the original paper, we have patients who dead within 30days and 180 days. Therefore, we classify patients who died within 28 days into the patients who died within 30 days. And the patients who died within 90 days and 180 days all are the category of 180 days. Survival patients as negative samples are the control group for all groups. Finally, through feature selection, feature vectorization, missing value handling, standardization and sample classification and other data processing procedures, we select 114 different samples for the experiment. The number of samples is shown in Table 5.

**Table 5. The number of samples for experiment**

| **Groups, alias** | **Positive samples{1}** | **Negative samples{0}** | **Total** |
| --- | --- | --- | --- |
| 1, W30D-H | 35 | 40 | 75 |
| 2, W180D-H | 19 | 20 | 39 |

The W30D-H dataset is the patients who died within 30 days or survived. The W180D-H dataset is the patients who died within 180 days or survived. And we apply our model to the data and gain the results as shown in Table 6 and Fig 10.

**Table 6. The results in HFP-ZGFPH database**

| **Evaluation** | **W30D-H (%)** | **W180D-H (%)** |
| --- | --- | --- |
| ACC | 66.67 | 58.97 |
| PPV | 64.71 | 71.43 |
| NPV | 68.29 | 56.25 |
| Recall | 62.86 | 26.32 |
| F1 score | 63.77 | 38.46 |
| AUC | 70.50 | 46.00 |
| Micro-average AUC | 71.00 | 49.00 |

(a) (b)

**Fig 10. The AUC and Micro-average AUC in HFP-ZGFPH database.**

We use seven criterions to measure the model. The PPV is positive prediction value and NPV is negative prediction value. And the PPV and NPV are recommended by the Reviewer #1: 3) comment. So when do this experiment, we also add those criterions. The formula of PPV and NPV is shown in Formula (1) and (2). Other criterions are both from the section of Training strategy in original paper. Fig 10 displays the AUC and Micro-average AUC. The left figure is W30D-H result. Another is W180D-H. In order to match the features of the original data, the database has 24 features been added digit 0. Moreover there are other features like age and heart rate replaced by approximations or other features. Therefore, the overall effect of the model is not as good as the effect of the test set in the original experiment, especially the W180D-H dataset.

The PPV in W180D-H is higher than other criterions, which means that most of the samples that are predicted to be positive are actually positive samples. The database does not report whether patients died or alive over a longer period of time. In other word, there is no record of death within a year or longer year. Therefore, the survival patients may similar than the patients died within 180 days, which may account for the lower NPV and lower F1 scores.

On account of data defects we from other point to deal with over-fitting. In our experiment we use the 5 fold cross-validation to overcome overfitting. The process of 5 fold cross-validation used in the original paper is shown in Fig 11. In our study, to begin with we divide the dataset into training data (95%) and test data (5%). We continue to divide the training data into 5 smaller sets. And proposed model is trained using 4 of the folds as training data. The above process is iterated five times. Then, we obtain the best model from the 5 times training. At last, we apply the model to the test data. From Fig 11, we know that test data do not participate in the whole training process and it can be used as external validation. The model performance from the test data is the final performance of proposed model. In addition, all the results shown in the original paper are from test data.

**Fig 11. The process of 5 fold cross-validation in this study**

Reviewer #1: 3)

In order to understand the results more accurately, it is better to provide PPV, NPV of the model.

This comment mainly involves the section of *Training strategy.* In the section of *Training strategy*, we introduce five criterions to measure our model. There are accuracy, recall, F1 score, AUC and Micro-average AUC. The accuracy is measured how many of the samples are predicted correctly. Because of the imbalanced problem, we use recall and F1 score to measure the effectiveness of the model. The recall reflects the proportion of positive classes in the true positive class. Furthermore, we set the death people as positive sample, so the model’s goal is to try to find as many positives as possible from the sample of real positives. So Recall is an important standard. The precision is positive prediction value also named PPV, which is the ratio of true positive samples to predict positive samples. And F1 score is a weighted average of precision and Recall, reflecting the whole performance of the model synthetically. Because we have shown the model performance using recall and F1 score, we do not display the model PPV results in tables and figures. Considering the datasets are imbalanced, the model stability is crucial. So in addition to AUC, we calculate the micro-average and macro-average of the AUC. Compared with the macro-average AUC, micro-average is more affected by the small samples. In our study, small samples both are positive samples. Hence, the micro-average of AUC under consideration is better reflecting stability.

Table 7 is the proposed model’s performance with two columns of data added, PPV and NPV.

**Table 7. The proposed model’s performance.**

| **Datasets** | **ACC** | **PPV** | **NPV** | **Recall** | **F1** | **AUC** | **Micro-average AUC** |
| --- | --- | --- | --- | --- | --- | --- | --- |
| W30D | 84.56 | **76.28** | **83.57** | 81.26 | 78.69 | 91.00 | 91.00 |
| W180D | 82.08 | **59.94** | **85.21** | 53.13 | 56.33 | 82.00 | 88.00 |
| W365D | 88.56 | **31.03** | **94.01** | 38.46 | 34.35 | 75.00 | 91.00 |
| A365D | 76.07 | **47.35** | **78.14** | 45.37 | 46.34 | 73.00 | 93.00 |

We know that PPV is the positive prediction value, which is the same as precision indicator. The formula is shown in Formula (1). And NPV is the negative prediction value, shown in Formula (2). Because in our data, we define the dead patient is positive sample and alive patient is negative sample. And the number of the positive sample is much smaller than the negative sample. So, the NPV value is much higher than the PPV shown in Table 7. In other words, NPV value is higher than PPV, which is normal because of data imbalance problem. And that is the reason why the original paper did not consider the NPV value to understand the model.

$PPV=Precision=\frac{TP}{TP+FP}$	(1)

$NPV=\frac{TN}{TN+FN}$	(2)

On the contrary, because of the imbalance problem, we pay more attention on the Recall, F1 score and AUC, which are applied to measure the model's ability to deal with imbalances and model stability. Although, the model’s F1 score in W365D is lowest. The model could balance PPV and Recall as much as possible. The predictive power of the model for negative samples is ignored. Taking the advice of this comment, we add PPV and NPV as the other two criteria to the existing criteria. Finally, there are four tables with two rows of data added, PPV and NPV.

Table 8 in this paper is Table 8 in the original paper. Table 8 is showing the different effect of adding indicator vector or not. The better results in Table 8 are bold. On the whole, the effect of using the indicator vector is better than not using indicator vector shown in Table 8. In W30D database, the imbalance rate is lowest. So the F1 score is highest. Adding indicator helps model to identify more positive samples from true positive samples. Furthermore the NPV value is higher than not adding the indicator vector which indicates the model can comprehensively handle the positives and negative samples.

**Table 8. Effects before and after adding indicator vector**

| **Datasets** | **Measurement** | **Before** | **After** | **Datasets** | **Measurement** | **Before** | **After** |
| --- | --- | --- | --- | --- | --- | --- | --- |
| **W30D** | ACC | 81.64 | **84.56** | **W365D** | ACC | 86.72 | **88.56** |
|  | PPV | **82.49** | 76.28 |  | PPV | **33.33** | 31.03 |
|  | NPV | 79.24 | **83.57** |  | NPV | **94.37** | 94.01 |
|  | Recall | 68.32 | **81.26** |  | Recall | 17.64 | **38.46** |
|  | F1 | 74.74 | **78.69** |  | F1 | 23.08 | **34.35** |
|  | AUC | 79.24 | **91.00** |  | AUC | 61.46 | **75.00** |
|  | Micro-average AUC | 90.00 | **91.00** |  | Micro-average AUC | **92.00** | 91.00 |
| **W180D** | ACC | 80.47 | **82.08** | **A365D** | ACC | 70.37 | **76.07** |
|  | PPV | 56.69 | **59.94** |  | PPV | 46.92 | **47.35** |
|  | NPV | 84.94 | **85.21** |  | NPV | 76.62 | **78.14** |
|  | Recall | 51.14 | **53.13** |  | Recall | 43.49 | **45.37** |
|  | F1 | 53.77 | **56.33** |  | F1 | 45.14 | **46.34** |
|  | AUC | 71.61 | **82.00** |  | AUC | 62.76 | **73.00** |
|  | Micro-average AUC | 88.00 | **88.00** |  | Micro-average AUC | 77.00 | **93.00** |

In W365D database, the imbalance rate is highest reaching 7.9981. The negative samples far outnumber positive samples. Therefore, the F1 is lowest. But Recall in adding indicator vector is higher than not adding. Also due to highest imbalance rate, NPV is highest in four types of data. By comparing the F1 score, Recall and PPV value of the W365D dataset, it can be seen that adding indicator vectors can help the model balance positive and negative sample prediction. And misdiagnosis rate is lower than not adding indicator vector, which can be obtained from the comparison of PPV value.

Table 9 in this paper is Table 9 in the original paper. Table 9 is showing the different effect of using attention or not. Because PPV value and Recall are mutually impacted. Recall in DLS-MSM with attention mechanism is higher than without attention. On the contrary, PPV value in without attention model is higher than with attention. This result involves the problem of the recognition rate. The higher Recall means that the model can distinguish true positive samples. The NPV value reflects that the DLS-MSM with attention can accurately identify negative samples. In addition, the model with attention is more stable, which is displayed by the AUC and Micro-average AUC.

**Table 9. Effect of using attention and not using**

| **Position** | **Measurement** | **W30D** | **W180D** | **W365D** | **A365D** |
| --- | --- | --- | --- | --- | --- |
| **Without attention** | ACC | 82.51 | 80.75 | 81.79 | 74.29 |
|  | PPV | 75.04 | **66.27** | **31.24** | **54.72** |
|  | NPV | **86.78** | 78.10 | 93.53 | 73.73 |
|  | Recall | **81.35** | 43.51 | 34.19 | 37.54 |
|  | F1 | 78.07 | 52.53 | 32.65 | 44.53 |
|  | AUC | 81.42 | 74.90 | 60.68 | 66.78 |
|  | Micro-average AUC | 90.00 | 89.00 | 90.00 | 82.00 |
| **DLS-MSM**  **(With attention)** | ACC | **84.56** | **82.08** | **88.56** | **76.07** |
|  | PPV | **76.28** | 59.94 | 31.03 | 47.35 |
|  | NPV | 83.57 | **85.21** | **94.01** | **78.14** |
|  | Recall | 81.26 | **53.13** | **38.46** | **45.37** |
|  | F1 | **78.69** | **56.33** | **34.35** | **46.34** |
|  | AUC | **91.00** | **82.00** | **75.00** | **73.00** |
|  | Micro-average AUC | **91.00** | **88.00** | **91.00** | **93.00** |

Table 10 in this paper is Table 10 in the original paper. Table 10 is showing model performance compared with other methods. We compare DLS-MSM model with six machine learning algorithms shown in Table 10. The other machine learning algorithms except DLS-MSM have their advantages especially for the W30D dataset. In W30D dataset, the Random Forest (RF) algorithm has the highest values in ACC, NPV, Recall and Micro-average AUC. It can be seen from this phenomenon that the ability of RF to process W30D is no less than DLS-MSM. And Support Vector Machine (SVM) is another well resulted machine learning algorithms. F1 score is highest in W30D database.

However as the imbalance rate increases and the sample differentiation decreases, the effect of DLS-MSM becomes apparent. DLS-MSM can deal with the problem of imbalance. In W365D database, F1 score of SVM and RF is null values. It shows RF and SVM both have difficulty dealing with imbalanced data. Multi-layer perception (MLP) and Light Gradient Boosting Machine (LigthGBM, LGB) are same as RF and SVM. The NPV in the four algorithms are basically flat at 99.88%, which indicates algorithms are hard to solve the imbalance problem. In this point, the DLS-MSM with highest PPV and F1 score is much better than other algorithms.

**Table 10. Model performance compared with other methods**

| **Datasets** | **Measurement** | **DLS-MSM** | **SVM** | **MLP** | **LR** | **RF** | **LGB** | **KNN** |
| --- | --- | --- | --- | --- | --- | --- | --- | --- |
| **W30D** | ACC | 84.56 | 85.12 | 80.95 | 82.44 | **86.31** | 84.23 | 80.35 |
|  | PPV | 76.28 | **80.64** | 70.16 | 70.96 | 72.58 | 77.40 | 64.52 |
|  | NPV | 83.57 | 87.74 | 83.04 | 89.15 | **94.34** | 88.21 | 89.62 |
|  | Recall | 81.26 | 79.37 | 76.32 | 79.28 | **88.24** | 79.34 | 78.43 |
|  | F1 | 78.69 | **80.00** | 73.11 | 74.89 | 79.64 | 78.36 | 70.80 |
|  | AUC | **91.00** | 83.97 | 79.82 | 81.64 | 86.85 | 83.15 | 79.82 |
|  | Micro-average AUC | 91.00 | 90.00 | 89.00 | 90.00 | **92.00** | 91.00 | 89.00 |
| **W180D** | ACC | **82.08** | 80.14 | 76.60 | 79.79 | 79.08 | 78.72 | 80.85 |
|  | PPV | **59.94** | 32.86 | 47.30 | 34.28 | 20.00 | 35.71 | 34.29 |
|  | NPV | 85.21 | 95.75 | 83.32 | 94.81 | **98.58** | 92.92 | 96.23 |
|  | Recall | 53.13 | 71.86 | 51.33 | 68.57 | **82.35** | 62.50 | 75.00 |
|  | F1 | **56.33** | 45.10 | 49.23 | 45.71 | 32.18 | 45.45 | 47.06 |
|  | AUC | **82.00** | 76.54 | 68.31 | 74.97 | 80.61 | 71.95 | 78.30 |
|  | Micro-average AUC | **88.00** | 85.00 | 85.00 | **88.00** | 86.00 | **88.00** | 86.00 |
| **W365D** | ACC | 88.56 | 88.70 | 87.87 | 88.70 | 88.70 | **89.12** | 88.28 |
|  | PPV | **31.03** | 0.00 | 18.52 | 3.71 | 0.00 | 3.7 | 0.00 |
|  | NPV | 94.01 | 100.0 | 95.75 | **99.53** | 100.0 | 100.0 | 99.53 |
|  | Recall | 38.46 | 0.00 | 41.67 | 50.00 | 0.00 | **100.0** | 0.00 |
|  | F1 | **34.35** |  | 25.64 | 6.90 |  | 7.14 | 0.00 |
|  | AUC | 75.00 |  | 65.99 | 69.51 |  | **93.53** | 44.32 |
|  | Micro-average AUC | 91.00 |  | 91.00 | **93.00** |  | 92.00 | 90.00 |
| **A365D** | ACC | **76.07** | 71.96 | 70.94 | 76.01 | 72.97 | 74.66 | 72.63 |
|  | PPV | **47.35** | 9.52 | 35.71 | 30.96 | 4.76 | 29.76 | 22.62 |
|  | NPV | 78.14 | 96.70 | 83.08 | 93.87 | **100.0** | 92.45 | 92.45 |
|  | Recall | 45.37 | 53.33 | 43.42 | 66.67 | **100.0** | 60.98 | 54.29 |
|  | F1 | **46.34** | 16.16 | 39.19 | 42.28 | 9.09 | 40.00 | 31.93 |
|  | AUC | 72.00 | 63.14 | 63.08 | 72.05 | **86.30** | 68.92 | 64.69 |
|  | Micro-average AUC | **93.00** | 74.00 | 76.00 | 82.00 | 82.00 | 82.00 | 77.00 |

Besides, DLS-MSM has the ability to solve similar problems with positive and negative samples. A365D dataset contains the dead patients who are dead after one year and even dead after five years as positive samples. And the negative samples are alive in the ten years of statistics. Therefore, positive samples and negative samples have higher similarity than other datasets. But DLS-MSM compared with other algorithms has highest ACC, PPV and F1 score. It indicates that the proposed model can recognize true positive samples from similar negative samples. All in all DLS-MSM shows a better result by contrast with other algorithms.

In the original paper, the multi-head self-attention has five positions in DLS-MSM framework. In Fig 12, the star is used to reveal the multi-head self-attention’s location. Because the input and the first fully connected layer are one-dimensional, according to the formula (3) shown as follows, the score of attention is a number instead of a vector, which is invalid for the attention mechanism.

$attention(Q,K,V)=softmax(\frac{QK^{T}}{\sqrt{d\_model}})\times V$	(3)

The formula (3) is from the original study. The Q, K and V mean the input from last structural block. d_model is the input dimension.

**Fig 12. The multi-head self-attention is in different locations in DLS-MSM**.

Therefore, we choose second position and third position to make a comparison. Table 11 in this paper is Table 11 in the original paper. Table 11 is showing the effects of different positions of multi-head self-attention. PPV value in position 2 and position 3 is average 0.5413% higher than position 4. This introduces that the position 2 and 3 have little effect on improving model performance. However the NPV in position 4 is average 1.966% higher than position 2 and 3. It indicates the model with attention in position 4 can effectively judges negative samples. Most other indicators results in position 4 are better than the other two positions. The results confirm the validity of the idea that attention is applied after the splicing layer.

The above is the specific analysis of PPV and NPV. Because of the mutual [effect](javascript:;) of PPV and Recall, the F1 score is introduced to systematically analyze model performance. On account of the imbalanced data, the NPV value is always high. And only from the NPV value, work well in this model.

**Table 11. The effects of different positions of multi-head self-attention**

| **Position** | **Measurement** | **W30D** | **W180D** | **W365D** | **A365D** |
| --- | --- | --- | --- | --- | --- |
| **Position 2** | ACC | 80.78 | 76.35 | **89.43** | 66.28 |
|  | PPV | **76.94** | 51.79 | **32.53** | 38.86 |
|  | NPV | 83.04 | 81.19 | 92.49 | 76.92 |
|  | Recall | 71.11 | 49.62 | 11.97 | 39.48 |
|  | F1 | 73.91 | 50.68 | 17.50 | 39.17 |
|  | AUC | 79.91 | 67.84 | 60.31 | 57.88 |
|  | Micro-average AUC | 89.00 | 84.00 | **91.00** | 73.00 |
| **Position 3** | ACC | 79.37 | 77.66 | 83.55 | 73.93 |
|  | PPV | 74.18 | 55.39 | 31.83 | **63.35** |
|  | NPV | 81.94 | 82.35 | 91.18 | 77.02 |
|  | Recall | 70.70 | 45.04 | 23.93 | 12.30 |
|  | F1 | 72.40 | 49.68 | 27.32 | 20.60 |
|  | AUC | 78.27 | 69.30 | 60.47 | 68.93 |
|  | Micro-average AUC | 87.00 | 85.00 | 90.00 | 81.00 |
| **Position 4**  **(DLS-MSM)** | ACC | **84.56** | **82.08** | 88.56 | **76.07** |
|  | PPV | 76.28 | **59.94** | 31.03 | 47.35 |
|  | NPV | **83.57** | 85.21 | **94.01** | **78.14** |
|  | Recall | **81.26** | **53.13** | **38.46** | **45.37** |
|  | F1 | **78.69** | **56.33** | **34.35** | **46.34** |
|  | AUC | **91.00** | **82.00** | **75.00** | **73.00** |
|  | Micro-average AUC | **91.00** | **88.00** | **91.00** | **93.00** |

Reviewer #1: 4)

This study did not present the left ventricular function of the study population. In the study of heart failure, absence of LV ejection fraction data is a critical limitation to claim clinical value of the study. At this point, this study seems more suitable for a computer science journal than a medical journal.

Thank you very much for this comment. In response to this comment, we have a discussion with our partner hospital’s doctors. And we learn that the LV ejection fraction refers to stroke output as a percentage of ventricular end-diastolic volume. Ejection fraction is related to the contractility of myocardium. The stronger the contractility of myocardium, the more output per stroke and the larger the ejection fraction. Conversely, low ejection fraction reflects weak systolic ability of the heart. Clinically, patients with heart failure often show a low ejection fraction. Therefore, ejection fraction is an important indicator to measure whether a patient has heart failure or not. However the research object in our study is the patient who has been diagnosed with heart failure. Their ejection fraction tends to be at abnormal levels. Therefore, considering ejection fraction has less practical point. Moreover, our attention is on the features which impact on the mortality of heart failure patients. The heart failure patients also have many complications. Our study is based on a comprehensive of features, not only ejection fraction.

To be honest, at the beginning of the study we consider that LV ejection fraction should be extracted from the MIMIC-III database which supports the experiment in this study. And we also want to make the LV ejection fraction as one of the characteristics for predicting the mortality of heart failure patients. However 10311 patients have very small amount of LV ejection fraction data. In MIMIC-III, the LV ejection fraction is not existed. But the database contains the ejection fraction in the *d_items* table which is a two-dimensional table in MIMIC-III. And the serial number of ejection fraction is 227008 as shown in Fig 13. We combine the heart failure patients’ serial number and ejection fraction number to find the laboratory test value from *chartevents* table which is also a two-dimensional table in MIMIC-III. At last, we totally obtain 2 pieces of ejection fraction, representing less than 0.02% of the total data. The data missing rate is extremely high.

Because of the above two points, we give up the idea using the ejection fraction as an indicator for mortality prediction.

**Fig 13. Result of selecting the ejection fraction item id**

We think our study is suitable for this journal. There are three reasons as follows.

First, our study is within the scope of submission to this journal. Our study is based on biomedical and computer science. And we mainly use the neural network to solve a medical problem how to analyze the mortality of the heart failure patient based on many features by computer. The scope of this journal contains medical research and engineering and this journal also welcome to interdisciplinary research, which can be found from website https://journals.plos.org/plosone/s/journal-information#loc-scope. From this point, our study is suitable for this journal.

Second, previous articles published in this journal have also included studies on the intersection of medical research and computer science. For example, Xiaolong Hu at el.^[3]^ proposed a hierarchical dilation convolutional network named HDC-Net for extracting blood vessels information. Constructing the deep learning model helps doctors to make early treatment and prevention of ophthalmic disease. This research has been published in volumes 16(9), pages 1-20, September, 2021 of this journal. And this research is same as our study domain, both on the intersection of medical research and computer science. D Wang et al.^[4]^ used a combination of deep features and machine learning classification to diagnose infected COVID-19 patients from chest X-ray. This is another example that proves this journal welcomes researches on the intersection of medical research and computer science. In particular, a paper named Artificial intelligence algorithm for predicting mortality of patients with acute heart failure has been published in volumes 14(7), 2019 of this journal. This study also cited by us in the original paper and it focuses on proposing computer models to make a mortality prediction for acute heart failure patients^[5]^. This is same as our study. From this point, our study is suitable for this journal.

Third, other medical or biomedical journals also accept some research on the intersection of medical research and computer science. For example, the journal named Journal of Heart and Lung Transplantation (JHLT) published a research called Artificial Intelligence Helps Predict 5-year Mortality and Graft Failure in Patients Undergoing Orthotopic Heart Transplantation. Agasthi P et al.^[6]^ used machine learning algorithm to predict survival and graft failure 5 years after orthotopic heart transplantation. We think the reason why a medical journal accepts the papers on the combination of medical research and computer science is the journal wants to handle some medical problems by computer models. And we think this journal may have the same original intention, using a computer model to assist therapy. A mortality prediction model based on deep learning model can help the doctor make more scientific treatment plans, which is our [research](javascript:;) [significance](javascript:;). From this point, our study is suitable for this journal.

Reviewer #2: 1)

The introduction is too long - it could be made significantly shorter by focussing on the role of ML mortality prediction in HF.

This comment mainly involves the section of *Introduction.* In the section of *Introduction* there are four parts. First, we introduce the significance of predicting the mortality of HF patients. Second we introduce the traditional mortality prediction model. Third we introduce the mortality prediction systems based on machine learning and deep learning algorithm. And at last we explain the shortcomings of the current model and the main research content of original paper.

After being reminded by this comment, we realize that the traditional medical model applied in mortality prediction is not relevant to this paper. The traditional prognostic mortality prediction model of heart failure patients uses the collected data and test results to find objective evidence based on the medical perspective. And then the model uses experience and some statistical methods to obtain the risk score or death results of heart failure patients. Therefore after taking the comment of the reviewer into consideration, we decide to delete the second part. That is to say, we delete the introduction about the traditional mortality prediction in order to focus on the machine learning and deep learning algorithm. In addition, we condense the research significance of the first part and revise the third part of the introduction. References and the order of references are also changed.

Furthermore in original paper, we mark the related content and use the style “~~a delete sentence~~” to delete relevant content. And the original reference has been deleted and new references are labeled by using the style “the original part~~[1]~~[2]”.

Reviewer #2: 2)

I was confused about how the patient population is split - it wasn't clear if subjects could be a more than 1 group - please clarify maybe with a flow diagram.

This comment mainly involves the section of *Data extraction*. In this paper, we make four binary classifications. Subjects couldn’t be a more than 1 group. More descriptions are as follows.

Under the criterion that age greater than or equal to 18 years, we extracted 10311 heart failure (HF) patients in this paper. Starting point is defined as the time of first hospitalized HF patients, and the endpoint is the time when patients were dead or discharge. Then, each patient has a death time, such as 0, 364. Zero means a patient is alive in the statistical period. The number 364 means that a patient died 364 days after being admitted to the hospital for the first time. So, we divided patients by death time. The flow diagram is as followed.

**Fig 14. Divide the patient category diagram according to the death time.**

As shown in the Fig 14, patients are divided into five categories: survivable patients who are alive in the statistical period, dead within 30 days patients, dead within 180 days patients, dead within 365 days patients and patients died after 365 days. Each group of died patients formed a group with those who did not. Therefore, in this paper totally compose four binary experimental classifications. Every group data is a binary value {0, 1}. All patients who died were labeled as a positive sample, others are negative sample. Table 12 describes the details.

**Table 12. Group and number of experiments**

| **Groups, alias** | **Positive samples{1}** | **Negative samples{0}** | **Total** | **IR** |
| --- | --- | --- | --- | --- |
| 1, W30D | 2472, within 30 days patients | 4239, survivable patients | 6711 | 1.7148 |
| 2, W180D | 1393, within 180 days patients | 4239, survivable patients | 5632 | 3.0431 |
| 3, W365D | 530, within 365 days patients | 4239, survivable patients | 4769 | 7.9981 |
| 4, A365D | 1677, after 365 days patients | 4239, survivable patients | 5916 | 2.5277 |

Besides we calculate the imbalanced rate (IR) defined in the formula (4). Four datasets of imbalanced rate respectively is 1.7148, 3.0431, 7.9981 and 2.5277. The W365D dataset is the most imbalanced.

$IR=\frac{Total negative samples}{Total positive samples}$	(4)

Reviewer #2: 3)

The feature vectorisation needs more explanation - I think representative example of the feature vector (in supplemental information) would be very useful.

This comment mainly involves the section of *Feature vectorization*. For better explain feature vectorization, we have rewritten Table 3 and the original content related to Table 13. We replace Table 3 in the original paper with Table 13 shown as follows.

**Table 13. The total features of HF patients**

| **Index** | **Name** | **Representation** | **Meaning and scope** |
| --- | --- | --- | --- |
| 1 | Gender | One-hot coding, {0, 1} | 0 means male, 1 means female |
| 2 | Age | Value | 18-91.4 |
| 3-9 | Medication | One-hot coding, {0, 1} | 1 means used, 0 means not |
| 10-13 | Surgery | One-hot coding, {0, 1} | 1 means has had surgery, 0 means not |
| 14-39 | Related diseases | One-hot coding, {0, 1} | 1 means with this related diseases, 0 means not |
| 40 | ICU turnover times | Value | 0-41 |
| 41 | ICU stay time | Value | 0-268.25 |
| 42 | Stayed in CCU | One-hot coding, {0, 1} | 1 means stayed, 0 means not |
| 43-86 | Laboratory test | Value and one-hot coding, {0, 1} | — |
| 87-88 | Heart rate | Value and one-hot coding, {0, 1} | 30-303 |
| 89-90 | BMI | Value and one-hot coding, {0, 1} | 11.7-78.4 |

In order to describe the feature vectorization more clearly, we take the patient id is 9 for an example. The patient information and meaning are shown in Table 14. Table 14 shows specific examples of nine types of feature information. There are 39 rows discrete features and 27 row continuous features. Because total 24 continuous features such as laboratory test, heart rate and BMI need to be processed by missing padded method, which is proposed in the section of *Proposed indicator vector for missing values method*. Therefore there are totally **90** features. The composition of features is shown in Table 15.

**Table 14. An example for describing the feature vectorizaion**

| **Index** | **Category** | **Name** | **Representation** | **Meaning** |
| --- | --- | --- | --- | --- |
| 1 | Gender | Gender | 0 | Male |
| 2 | Age | Age | 41.7902 | — |
| 3 | Medications | ACEI | 1 | Used the medicine |
| 4 |  | ARB | 0 | Not used the medicine |
| 5 |  | Beta-receptor blockers | 1 | Used the medicine |
| 6 |  | CCB | 1 | Used the medicine |
| 7 |  | Digitalis | 0 | Not used the medicine |
| 8 |  | Diuretic | 0 | Not used the medicine |
| 9 |  | Nitrates | 0 | Not used the medicine |
| 10 | Surgery | LVAD | 0 | Have not had surgery |
| 11 |  | CRT | 0 | Have not had surgery |
| 12 |  | ICD | 0 | Have not had surgery |
| 13 |  | Heart transplantation | 0 | Have not had surgery |
| 14 | Related diseases | Cardiac arrhythmias | 0 | Have not this disease |
| 15 |  | Valve disease | 0 | … |
| 16 |  | Endocarditis | 0 | Have not this disease |
| 17 |  | Pulmonary circulation disorder | 0 | Have not this disease |
| 18 |  | Respiratory failure | 0 | Have not this disease |
| 19 |  | Peripheral vascular disorder | 0 | Have not this disease |
| 20 |  | Hypertension | 1 | Have this disease |
| … |  | … | … | … |
| 39 |  | Abnormal heart structure | 0 | Have not this disease |
| 40 | ICU information | ICU turnover times | 1 | 1 times in ICU |
| 41 |  | ICU stay time | 5.3231 | 5.3231 days in ICU |
| 42 |  | Stayed in CCU | 0 | Not stay in CCU |
| 43 | Laboratory test | WBC | 18.6 | Value |
| 44 |  | Indicator vector-WBC | 0 | The above value is true |
| 45 |  | Hemoglobin | 14 | Value |
| 46 |  | Indicator vector-Hemoglobin | 0 | The above value is true |
| … |  | … | … | … |
| … |  | … | … | … |
| 85 |  | PCO2 | 67 | Value |
| 86 |  | Indicator vector-PCO2 | 0 | The above value is true |
| 87 | Heart rate | Heart rate | 111 | Value |
| 88 |  | Indicator vector-Heart rate | 0 | The above value is true |
| 89 | BMI | BMI | 31.09574 | Value |
| 90 |  | Indicator vector-BMI | 0 | The above value is true |

**Table 15. The composition of features**

| **Discrete features** | **Continuous features (27)** | | **Indicator vectors** | **Total features** |
| --- | --- | --- | --- | --- |
|  | Complete features | Missing features |  |  |
| 39 | 3 | 24 | 24 | **90** |

As the original article said, there are 39 discrete features coded by one-hot coding rule. And in Table 14, the gender is 0, which means the patient is male. Then in the medications, the ACEI, Beta-receptor blockers and CCB is 1, which means the patient has taken the three types of drug. On the contrary, the patient has not taken other drugs. Next in the surgery and related diseases, digit 1 means the patient has taken the corresponding surgery or has the related disease. For example, the patient in Table 14 has the hypertension. The feature of staying in CCU is in the same way.

As for other discrete features, the digit 0 and 1 act as indicator vector in missing value method. For example in Table 14 the Indicator vector-WBC is 0, which means the above WBC is true in the data and is not padded by us. The feature of heart rate and BMI is in the same way.

Reviewer #2: 4)

There is no need to discuss CNN architecture more generally only the 1D version.

This comment mainly involves the section of *CNN with different kernel sizes*. In order to highlight our CNN structure has only one dimension convolution, we discuss the main application scenarios of widely used 2D and 3D convolution. 2D convolution often is used in computer vision. And 3D is frequently applied in video processing field and medical CT image field. The 1D is often accepted in natural language processing and time-series data. The input in 1D convolution neural network is one dimension and from this point we adopt the 1D convolution not the 2D or 3D convolution.

Because we only use the 1D format, and as this comment said there is no need to discuss other types of convolution. We totally agree with the reviewer’s advice and delete the discussion about 2D and 3D convolution. We mark the related content and use the style “~~a delete sentence~~” to delete relevant content.

Reviewer #2: 5)

I was confused to how the 1x90 feature vector is created please clarify in relation to other earlier sections.

This comment mainly involves the section of *Proposed indicator vector for missing values method* and *CNN with different kernel sizes*. In the last paragraph of the section *Proposed indicator vector for missing values method*, we indicate the feature is 90. The reason that the dimension of the feature is 90 is clearly explained in the last two sentences of the second paragraph of part Reviewer #2:3 and the Table 4 in this paper also explains the reason. Although we use the one dimension convolution, the CNN model in **pytorch** framework needs the input is two dimensions. However the feature of HF patient is one dimension style. So we expand the original dimension and make it become 1*90. Therefore the input of CNN framework is 1*90 in the section *CNN with different kernel sizes*. Moreover in order to clarify in relation, we add an explanation marked yellow in the section *CNN with different kernel sizes* of the original paper.

Reviewer #2: 6)

Using a 1D CNN results in kernels crossing very different features - please discuss - why not use an MLP.

The essence of multi-layer perceptron (MLP) is the fully connection. However, the 1D CNN is local connection. Fig 15 and Fig 16 show the different connections. Local connection realized by convolution kernels lead to very different features, which is the original meaning of the comment. There are two reasons why we use local connection not fully connection.

In terms of CNN characteristics, the CNN effectively reduces the number of parameters and improves the learning speed compared with MLP. The essence of MLP is fully connection. And the CNN is local connection

**Fig 15. Multi-layer Perceptron**

As shown in Fig 15, the input is $\{x_{1},x_{2},\ldots,x_{6}\}$. And the layer is the 6*4 size. The output of layer is $\{y_{1},y_{2},\ldots,y_{4}\}$. Not considering the bias, the fully connection calculation formula is as follows. In one fully connection, there are 24 parameters.

$\left[ \begin{matrix} y_{1} \\ \begin{matrix} y_{2} \\ \begin{matrix} y_{3} \\ y_{4} \end{matrix} \end{matrix} \end{matrix} \right]=\left[ \begin{matrix} \begin{matrix} w_{11} & w_{12} & \begin{matrix} w_{13} & w_{14} & \begin{matrix} w_{15} & w_{16} \end{matrix} \end{matrix} \end{matrix} \\ \begin{matrix} w_{21} & w_{22} & \begin{matrix} w_{23} & w_{24} & \begin{matrix} w_{25} & w_{26} \end{matrix} \end{matrix} \end{matrix} \\ \begin{matrix} \begin{matrix} w_{31} & w_{32} & \begin{matrix} w_{33} & w_{34} & \begin{matrix} w_{35} & w_{36} \end{matrix} \end{matrix} \end{matrix} \\ \begin{matrix} w_{41} & w_{42} & \begin{matrix} w_{43} & w_{44} & \begin{matrix} w_{45} & w_{46} \end{matrix} \end{matrix} \end{matrix} \end{matrix} \end{matrix} \right]*\left[ \begin{matrix} x_{1} \\ x_{2} \\ \begin{matrix} x_{3} \\ x_{4} \\ \begin{matrix} x_{5} \\ x_{6} \end{matrix} \end{matrix} \end{matrix} \right]$	(4)

Fig 16 is the local connection process. The input is also $\{x_{1},x_{2},\ldots,x_{6}\}$. And the kernel size is 1*3. The stride is set to 1. Fig 16 includes four steps. After the local connection, the output is $\{y_{1},y_{2},\ldots,y_{4}\}$. Because CNN utilizes the strategy of parameter shared. The parameter is only 3. We can intuitively see the advantages of using CNN.

**Fig 16. Convolution process**

In our study, the dimension of feature is 90. Using the MLP is mean that we utilize all the information for predicting mortality. And the model must to integrate all the features to get the result. In other words, the MLP ignore the local spatial information. However, the CNN uses kernel to obtain local information. The local information maybe can derive the final prediction directly. And different combinations of features help the CNN to make more accurate prediction results.

**Table 16. The model consequence of MLP and DLS-MSM**

| **Datasets** | **Measurement** | **DLS-MSM** | **MLP** | **Datasets** | **Measurement** | **DLS-MSM** | **MLP** |
| --- | --- | --- | --- | --- | --- | --- | --- |
| **W30D** | ACC | 84.56 | 80.95 | **W365D** | ACC | 88.56 | 87.87 |
|  | Recall | 81.26 | 76.32 |  | Recall | 38.46 | 41.67 |
|  | F1 | 78.69 | 73.11 |  | F1 | 34.35 | 25.64 |
|  | AUC | 91.00 | 79.82 |  | AUC | 75.00 | 65.99 |
|  | Micro-average AUC | 91.00 | 89.00 |  | Micro-average AUC | 91.00 | 91.00 |
| **W180D** | ACC | 82.08 | 76.60 | **A365D** | ACC | 76.07 | 70.94 |
|  | Recall | 53.13 | 51.33 |  | Recall | 45.37 | 43.42 |
|  | F1 | 56.33 | 49.23 |  | F1 | 46.34 | 39.19 |
|  | AUC | 82.00 | 68.31 |  | AUC | 72.00 | 63.08 |
|  | Micro-average AUC | 88.00 | 85.00 |  | Micro-average AUC | 93.00 | 76.00 |

In terms of the final model consequence, the MLP is not better than CNN. In the original paper, we make a set of comparative experiments. We extract some experimental results as shown in Table 16. The DLS-MSM in Table 16 is the model proposed by us. There are five criterions to measure two models. The effect of MLP is inferior to DLS-MSM in four datasets. In particular, MLP has difficulty dealing with imbalanced data. The W365D is the most imbalanced data. The F1 score of MLP in 365D dataset is only 25.64% and smaller 8.71% than DLS-MSM.

Reviewer #2: 7)

Figure 3 seems to switch between showing inputs to kernels - maybe these should be more obviously differentiated.

This comment mainly involves the section of *CNN with different kernel sizes*. Due to CNN's excellent performance in feature extraction, we use the CNN structure in our paper. And because the features of patients are one dimension, the CNN structure is based on 1D convolution. Therefore, we draw the Figure 11, also based on 1D convolution. We combine Figure 3 and Figure 4 in the original paper, renamed Figure 11 as follows.

**Fig 17. The CNN based on multi-head self-attention for prediction.**

Figure 11 shows us the CNN structure adopted by us. We set the batch size is 256, which means the model trains 256 pieces of data at one time. And in response to Reviewer #2: 3), we explain the reason why the dimension is 90. In addition because the 1D convolution needs two dimensional input forms, so we expand the original one-dimensional feature dimension of patient. Therefore, the input is 1*90. Coupled with the batch size, the final input is 256*(1*90) which is the *Input* structure painted white on the far left of Figure 11.

Firstly the input goes through a set of one-dimensional convolution kernels which is the convolution layer structure painted purple second from the left in the Figure 11. The convolution layer consists of eight convolution kernels of the same size (1*3), batch normalization (BN) and ReLU activation function. Through the convolution layer, the output is 256*(8*90). Among them, BN is used to avoid the gradient disappearance and explosion phenomenon problems. The BN accelerates simultaneously the convergence rate.

Secondly after the convolution layer, the output becomes input and goes the different kernels structure, which is third from the left in the Figure 11. The structure consists of three different convolution groups. As shown in Figure 11, the red structure group contains three convolution kernels of the same size (1*3). And the blue structure group contains three convolution kernels of the same size (1*4). Then, the orange structure group contains three convolution kernels of the same size (1*5). After the input respectively goes through the convolution layer size with different convolution kernel size, the output becomes 256*(3*88), 256*(3*89) and 256*(3*88). Next, we add the BN, ReLU function and MaxPooling layer after each different convolution groups. Finally, through the different convolution groups, the input size respectively becomes 256*(3*44), 256*(3*44) and 256*(3*44).

Thirdly we stitch three different outputs from each different kernels structure, which named stitching layer in Fig 17. Therefore, the output changes to 256*(3*132) and 132 is to 44 (the red group) plus 44 (the blue group) plus 44(the orange group). Fourthly, we let the output pass the multi-head self-attention mechanism, which is between the stitching layer and fully connected structure. The head of the mechanism is set to 2. From the multi-head self-attention mechanism, the output maintains 256*(3*132).

**Fig 18. The CNN based on multi-head self-attention for prediction.**

Fifthly, we use fully connected layer, grey structure in Fig 18 which is the second to last on the right in the Figure 11. The fully connected layer structure contains two fully connected layers which are (396, 128) and (128, 2). The output from fully connected layer is 256*2. Finally, we obtain the final results from the output structure. Because the batch size is 256, we totally obtain 256 results. And in other words, we obtain 256 predictions on whether the patients are dead or alive.

The above is a detailed explanation of Figure 11. In order to be more detailed and obvious about the changes between kernels, we have changed the corresponding parts of the original paper. We replace Figure 3 in the original paper with Fig 18 above.

Reviewer #2: 8)

The effect of the indicator vector and self-attention head are in the discussion not the results please move to results.

This comment mainly involves the section of *Results* and *Discussion*. In the section of *Results*, we introduce the training strategy, mortality prediction for HF patients and model performance compared with other methods. And in the section of *Discussion*, we discuss the effect of the indicator vector, the effect of multi-head self-attention and important feature ranking based Deep SHAP. The effect of multi-head self-attention contains three parts. One is the difference of using attention and not using. The second is the different effects with different locations applied the multi-head attention. The last is the different effects with the different number of self-attention mechanism head.

After being reminded by this comment, we realize that the effect of indicator vector and attention should be suitably moved to the section of *Results*. Furthermore because using attention is a part of the CNN framework. Therefore, we break up the section of the effect of multi-head self-attention and move the subsection of the effect using attention into *Results.* In general after shifting, the section of *Results* includes the training strategy, mortality prediction for HF patients, the effect of indicator vector and the effect of using attention. And we discuss model performance compared with other methods, the different effects with different locations, the different influences with different number of self-attention mechanism heads and we also discuss important feature ranking based Deep SHAP. In the original paper, the changes are highlighted in yellow.

Reviewer #2: 9)

Depp SHAP is only really mentioned in the discussion - maybe more description in methods and results is required.

This comment is very thoughtful. Although Deep SHAP is used only in the section of *Discussion*, as the model that appears and is used in this study, it is necessary to describe it in more detail. Therefor we describe this model in more detail in the section of *Other models*.

Deep SHAP is an [interpretation](javascript:;) [model](javascript:;) for deep learning model and the model is proposed by Scott M. Lundberg and Su-In Lee in 2017^[7]^. Deep SHAP combines the DeepLIFT theory and SHAP value. DeepLIFT is used to interpret deep learning model by calculating the weight to each feature of the input in the backpropagation. And SHAP (SHapley Additive exPlanations) gives each feature a unique importance score. Deep SHAP is to calculate the SHAP value of a structure in the network to form the SHAP value of the entire network. Because the SHAP value can quickly analyze simple network structures, such as linear, max-min pooling, or activation functions with only one input. So the Deep SHAP can quickly gain the value of the whole model. Scott M. Lundberg and Su-In Lee combine the two theories to explain the deep learning model and prove it more suitable for human to understand model.

Deep SHAP is already wrapped in Python's package named shap. And in this study, we use the deepExplainer function in the shap package to get the weight for every feature. The weight results of the mortality prediction deep learning model are shown in the section of *Discussion*.

The above content is the response to every comment. We look forward to your reply.

Sincerely yours,

Dengao Li

References

1.	Zhang, Z., Cao, L., Zhao, Y., Xu, Z., Chen, R., Lv, L., & Xu, P. (2020). Hospitalized patients with heart failure: integrating electronic healthcare records and external outcome data (version 1.2). PhysioNet. <https://doi.org/10.13026/8a9e-w734>.

2.	Goldberger, A., Amaral, L., Glass, L., Hausdorff, J., Ivanov, P. C., Mark, R., ... & Stanley, H. E. (2000). PhysioBank, PhysioToolkit, and PhysioNet: Components of a new research resource for complex physiologic signals. Circulation [Online]. 101 (23), pp. e215–e220.

3.	Xiaolong Hu & Liejun Wang & Shuli Cheng & Yongming Li, 2021. "[HDC-Net: A hierarchical dilation convolutional network for retinal vessel segmentation](https://ideas.repec.org/a/plo/pone00/0257013.html)," [PLOS ONE](https://ideas.repec.org/s/plo/pone00.html), Public Library of Science, vol. 16(9), pages 1-20, September.

4.	Dingding Wang & Jiaqing Mo & Gang Zhou & Liang Xu & Yajun Liu, 2020. "[An efficient mixture of deep and machine learning models for COVID-19 diagnosis in chest X-ray images](https://ideas.repec.org/a/plo/pone00/0242535.html)," [PLOS ONE](https://ideas.repec.org/s/plo/pone00.html), Public Library of Science, vol. 15(11), pages 1-15, November.

5.	Kwon J-M, Kim K-H, Jeon K-H, Lee SE, Lees H-Y, Cho H-J, et al. Artificial intelligence algorithm for predicting mortality of patients with acute heart failure. Plos One. 2019;14(7). doi: 10.1371/journal.pone.0219302. PubMed PMID: WOS:000484939800028.

6.	Agasthi P, Smith SD, Murphy KM, Buras MR, Golafshar M, Herner M, et al. Artificial Intelligence Helps Predict 5-year Mortality and Graft Failure in Patients Undergoing Orthotopic Heart Transplantation. Journal of Heart and Lung Transplantation. 2020; 39(4): S142-S. PubMed PMID: WOS:000522637201022.

7.	Lundberg SM, Lee SI. A Unified Approach to Interpreting Model Predictions. In: Guyon I, Luxburg UV, Bengio S, Wallach H, Fergus R, Vishwanathan S, et al., editors. Advances in Neural Information Processing Systems 30. Advances in Neural Information Processing Systems. 30. La Jolla: Neural Information Processing Systems (Nips); 2017.
