# Supplementary material for: A deep learning system for heart failure mortality prediction
Source: PLoS One. 2023 Feb 24;18(2):e0276835. doi: 10.1371/journal.pone.0276835 (PMC9956019; doi:10.1371/journal.pone.0276835)
Supplement: Attachment — Submitted filename: Response to Reviewers.docx [file pone.0276835.s004.docx]

Dear editor,

Thank you very much for your valuable suggestions on our paper. We have complied with journal requirements and revised these opinions one by one at the first time. In the first revised manuscript with track changes file, the revision has been marked in yellow. And in the second revision, the changed part is marked in green. Please review the following the modification notes for each journal requirement and suggestion. The comment is blue and response is black.

Reviewer #2:1)

However I am still concerned about the use of a CNN for the network - I agree that CNN are easier to train and it does leverage local connections - but that is not always a good thing. For instance does the order of vector features matter - what happens when convolutional kernels cross different groups. I would suggest doing a sensitivity analysis of different order of features.

Following your advice, we did two different experiments with different order of feature to illustrate that the model is insensitive to feature order.

First of all because we used an indicator vector for missing values, which is illustrated in the subsection of *Proposed indicator vector for missing values method.* The indicator vector should not been separated by random. Therefore 24 features laboratory tests, heart rate and BMI together with their indicator vectors are considered as a whole. As shown in Table 1, the missing features(italic bold) and indicator vectors(italic bold) are a whole. Thence a total of 66 features are randomly shuffled.

**Table 1. The composition of features**

| Discrete features | Continuous features | | ***Indicator vectors*** | Total features |
| --- | --- | --- | --- | --- |
|  | Complete features | ***Missing features*** |  |  |
| 39 | 3 | 24 | 24 | 90 |

The feature order of these two experimental samples are shown in Tables 2 and 3.

**Table 2. First experimental sample**

| **Index** | **Name** | **Index** | **Name** | **Index** | **Name** |
| --- | --- | --- | --- | --- | --- |
| 41 | ICU stay time | 44 | Hemoglobin | 15 | Valve disease |
| 27 | Cardiomyopathy | 36 | Hyperthyroidism | 34 | Hyperlipidemia |
| 2 | Admission age | 24 | Alcohol abuse | 62 | Antithrombin |
| 3 | ACEI | 54 | Unitro | 33 | Diabetes |
| 21 | Renal disease | 14 | Cardiac arrhythmias | 55 | Kalium (K) |
| 56 | Sodium (Na) | 51 | [Aspartate](javascript:;) [aminotransferase](javascript:;) (AST) | 42 | Stayed in CCU |
| 58 | Hormothyrin (TSH) | 43 | White cells count (WBC) | 57 | Chloridion (Cl) |
| 39 | Abnormal heart structure | 52 | [Alkaline](javascript:;) [phosphatase](javascript:;) (ALP) | 12 | Automatic implantable cardioverter/defibrillator check (ICD) |
| 35 | Connective tissue disease | 29 | Coronary atherosclerosis I | 6 | CCB |
| 61 | Creatine kinase isoenzyme (CK-MB) | 22 | Renal disease | 38 | Other heart disease |
| 16 | Endocarditis | 1 | Gender | 50 | Alanine transaminase (ALT) |
| 18 | Respiratory failure | 9 | Nitrates | 30 | Coronary atherosclerosis II |
| 47 | Blood glucose (GLU) | 65 | Heart rate | 5 | Beta-receptor blockers |
| 25 | Drug abuse | 4 | ARB | 37 | Cardiac Arrest and Sudden Cardiac Death Disease |
| 28 | Myocarditis | 49 | Total bilirubin (TBIL) | 60 | Creatine kinase (CK) |
| 19 | Peripheral vascular disorder | 40 | ICU turnover times | 26 | Tobacco |
| 59 | ctni2 Troponin T (cTnT) | 20 | Hypertension | 8 | Diuretic |
| 23 | Obesity | 64 | [Arterial](javascript:;) [blood](javascript:;) [gas](javascript:;) [analysis](javascript:;) (pCO2) | 48 | [Albumin](javascript:;) (ALB) |
| 45 | Hematocrit | 63 | [Arterial](javascript:;) [blood](javascript:;) [gas](javascript:;) [analysis](javascript:;) (pO2) | 13 | Heart transplantation |
| 32 | Congenital cardiovascular disorders | 53 | [Serum](javascript:;) [creatinine](javascript:;) (CRE) | 46 | [Blood](javascript:;) [platelet](javascript:;) [count](javascript:;) (PLT) |
| 7 | Digitalis | 10 | Left ventricular assistant device (LVAD) | 31 | Pericardial disease |
| 17 | Pulmonary circulation disorder | 66 | BMI | 11 | Cardiac resynchronization therapy (CRT) |

Table 3. Second experimental sample

| **Index** | **Name** | **Index** | **Name** | **Index** | **Name** |
| --- | --- | --- | --- | --- | --- |
| 14 | Cardiac arrhythmias | 6 | CCB | 53 | [Serum](javascript:;) [creatinine](javascript:;) (CRE) |
| 60 | Creatine kinase (CK) | 40 | ICU turnover times | 45 | Hematocrit |
| 24 | Alcohol abuse | 36 | Hyperthyroidism | 58 | Hormothyrin (TSH) |
| 33 | Diabetes | 44 | Hemoglobin | 34 | Hyperlipidemia |
| 55 | Kalium (K) | 48 | [Albumin](javascript:;) (ALB) | 32 | Congenital cardiovascular disorders |
| 15 | Valve disease | 61 | Creatine kinase isoenzyme (CK-MB) | 9 | Nitrates |
| 7 | Digitalis | 51 | [Aspartate](javascript:;) [aminotransferase](javascript:;) (AST) | 31 | Pericardial disease |
| 4 | ARB | 20 | Hypertension | 42 | Stayed in CCU |
| 52 | [Alkaline](javascript:;) [phosphatase](javascript:;) (ALP) | 11 | Cardiac resynchronization therapy (CRT) | 47 | Blood glucose (GLU） |
| 27 | Cardiomyopathy | 5 | Beta-receptor blockers | 41 | ICU stays time |
| 38 | Other heart disease | 17 | Pulmonary circulation disorder | 59 | Troponin T (cTnT) |
| 35 | Connective tissue disease | 18 | Respiratory failure | 65 | Heart rate |
| 49 | Total bilirubin (TBIL) | 29 | Coronary atherosclerosis I | 57 | Chloridion (Cl) |
| 19 | Peripheral vascular disorderr | 28 | Myocarditis | 56 | Sodium (Na) |
| 26 | Tobacco | 37 | Cardiac Arrest and Sudden Cardiac Death Disease | 66 | BMI |
| 1 | Gender | 13 | Heart transplantation | 2 | Admission age |
| 46 | [Blood](javascript:;) [platelet](javascript:;) [count](javascript:;) (PLT) | 63 | [Arterial](javascript:;) [blood](javascript:;) [gas](javascript:;) [analysis](javascript:;) (pO2) | 50 | Alanine transaminase (ALT) |
| 62 | Antithrombin | 8 | Diuretic | 16 | Endocarditis |
| 22 | Renal failure | 39 | Abnormal heart structure | 30 | Coronary atherosclerosis II |
| 25 | Drug abuse | 10 | Left ventricular assistant device (LVAD) | 64 | [Arterial](javascript:;) [blood](javascript:;) [gas](javascript:;) [analysis](javascript:;) (pCO2) |
| 23 | Obesity | 21 | Renal disease | 54 | [Urea](javascript:;) [nitrogen](javascript:;) (BUN) |
| 3 | ACEI | 12 | Automatic implantable cardioverter/defibrillator check (ICD) | 43 | White cells count (WBC) |

We used the order of data in Table 2 and 3 to predict mortality and we also divided data into five categories: survivable patients who are alive in the statistical period, dead within 30 days patients, dead within 180 days patients, dead within 365 days patients and patients died after 365 days. Each group of died patients form a group with those who did not, which is the same as the original paper. After predicting, we gained the results in Table 4. The word **One** in the Table 4 is based on first experimental sample and the word **Second** is based on second experimental sample. The word **Original** is mean that the results is from original paper.

**Table 4.The prediction results**

| **Datasets** | **Measurement** | **One** | **Second** | **Original** | **Datasets** | **Measurement** | **One** | **Second** | **Original** |
| --- | --- | --- | --- | --- | --- | --- | --- | --- | --- |
| W30D | ACC | 83.92 | 85.01 | 84.56 | W365D | ACC | 88.32 | 88.79 | 88.56 |
|  | PPV | 76.44 | 75.49 | 76.28 |  | PPV | 30.99 | 31.45 | 31.03 |
|  | NPV | 82.76 | 83.21 | 83.57 |  | NPV | 93.85 | 93.96 | 94.01 |
|  | Recall | 81.05 | 80.98 | 81.26 |  | Recall | 38.54 | 38.98 | 38.46 |
|  | F1 | 78.64 | 78.34 | 78.69 |  | F1 | 34.21 | 34.64 | 34.35 |
|  | AUC | 91.00 | 91.00 | 91.00 |  | AUC | 75.00 | 75.00 | 75.00 |
|  | Micro-average AUC | 90.00 | 90.00 | 91.00 |  | Micro-average AUC | 91.00 | 91.00 | 91.00 |
| W180D | ACC | 82.12 | 82.72 | 82.08 | A365D | ACC | 76.15 | 75.86 | 76.07 |
|  | PPV | 59.74 | 59.65 | 59.94 |  | PPV | 47.21 | 47.90 | 47.35 |
|  | NPV | 86.23 | 85.56 | 85.21 |  | NPV | 78.12 | 77.93 | 78.14 |
|  | Recall | 52.94 | 53.10 | 53.13 |  | Recall | 45.18 | 44.97 | 45.37 |
|  | F1 | 56.61 | 56.20 | 56.33 |  | F1 | 45.89 | 46.48 | 46.34 |
|  | AUC | 81.00 | 82.00 | 82.00 |  | AUC | 73.00 | 73.00 | 73.00 |
|  | Micro-average AUC | 87.00 | 88.00 | 88.00 |  | Micro-average AUC | 93.00 | 93.00 | 93.00 |

From results of the Table 4, we found the order of vector features did not matter the model prediction, when convolutional kernels cross different groups. And that is to say, the model is not sensitive to different order of features.

Reviewer #2:

Also please put the comparison with different ML models in the results not discussion.

This comment mainly involves the section of *Results* and *Discussion*. In the section of *Results*, we introduce the training strategy, mortality prediction for HF patients, the effect of indicator vector and the effect of using attention. And in the section of Discussion, we discuss the model performance compared with other methods, the effect of attention in different locations and having different number of heads and the important feature ranking based Deep SHAP.

After being reminded by this comment, we realize that the model performance compared with other methods should be suitably moved to the section of *Results*. Therefore in the section of *Results,* there are five parts the training strategy, mortality prediction for HF patients, the model performance compared with other methods, the effect of indicator vector and the effect of using attention. In the original paper, the changes are highlighted in green.

Reviewer #2:3)

The paper is still very long and needs to be edited to make it shorter.

After your reminder, I found that the article has some redundant and unnecessary statements in some places. After the revision, the number of words in the article including author introduction, references and other structures has been reduced from 9506 words to 7307 words. On the basis of maintaining the meaning of the original paper, we have deleted about 23.12% of the paper and mainly modified five parts. The main reasons are as follows.

First we edited the section of *Data preprocessing.* In the subsection of *Feature vectorization,* we describe in detail how each feature is vectorized. However each classification of feature is concisely shown in Table 3 in the original paper. Therefore we simplified the detailed introduction about the feature vectorization processing. In the subsection of *Proposed indicator vector for missing values method*, we simplified description of data imputation theory. And a direct explanation of the filling method, Mean with variance, is not applicable. In addition, laboratory tests are also shown in Table 3 in original paper. Therefore, we simplified description of laboratory variables. Z-score normalization method is a common standardization method. Hence, we assume that readers will be familiar with this method and therefore do not repeat it and delete this subsection.

Second in the subsection of *Deep learning model*, we introduced the other models compared with our framework. There are Support Vector Machine (SVM), Multi-layer Perceptron (MLP), Logistic Regression (LR), Random Forest (RF), Light Gradient Boosting Machine (LigthGBM, LGB) and K-Nearest Neighbor(KNN). Because those models are representative and widely applied in the medical field or bio-medical field. Thus there is no need to introduce those models specifically. Therefore we deleted the subsection of *Other Models* and made a brief introduction where it appears. And it is briefly described in the subsection of *Model performance compared with other methods*.

Third we edited the section of *Results.* In the subsection of *Training strategy*, we simplified the introduction to model training parameters and the introduction to model evaluation metrics.

Fourth we revised the section of *Discussion.* In the subsection of *Effect of attention in different locations,* we detailed the five different positions in which the attention can exist and explained why we only chose position 2 and position 3 as comparative experiments. However, as the above said the comparative experiment only involved the second position and the third position. There is no need to describe other locations in great detail. Thus, we have deleted the second and third paragraph of this chapter and put the important sentence into first paragraph. In addition to, we simply stated the reason why we used the position 2 changed to position 1 and position 3 changed to position 2 in the original paper. Furthermore, we have simplified the description and interpretation of the results of the trials at different positions.

Fifth we modified repeated express all over the paper. An example in the subsection of *Effect of different number of heads* is as follows.

The original sentence is as follows. The multi-head self-attention mechanism can have different heads. Hence, the different numbers of the head influence the system performance.

After being edited, the sentence is as follows. The multi-head self-attention mechanism can have different heads, which may influence the system performance.

Furthermore in original paper, we mark the related content and use the style “~~a delete sentence~~” to delete relevant content. And the original reference has been deleted and new references are labeled by using the style “the original part~~[1]~~[2]”.

Reviewer #3:

Publish the revised manuscript as I am satisfied with the changes made by authors. I would like to appreciate the efforts made by authors in preparing the revised version.

Thanking you very much for your recognition. It is my honor that the reviewer agrees with my revision.

The above content is the response to each comment. We look forward to your reply.

Sincerely yours,

Dengao Li
